# Research trends and hotspots in the surgical treatment of peripheral nerve injuries of the upper limb from 2000 to 2024: a bibliometric visualization study

**DOI:** 10.3389/fneur.2025.1463080

**Published:** 2025-02-13

**Authors:** Jian Ruan, Hekun He, Xueyuan Li, Hong Chen

**Affiliations:** Department of Hand Surgery, Ningbo No. 6 Hospital, Ningbo, China

**Keywords:** upper limb, peripheral nerve injuries, surgery, bibliometric analysis, CiteSpace

## Abstract

**Purpose:**

Surgical treatment plays a crucial role in the management of peripheral nerve injuries of the upper limb, but little bibliometric analysis has been conducted on it. This study was aimed to examine the global trends and hotspots in the field of Peripheral nerve injuries of the upper limb.

**Methods:**

Publications on the surgical treatment of peripheral nerve injuries of the upper limb in the Web of Science database were collected between 2000 to 2024. CiteSpace and VOSviewer software was applied to visualize and analyze publications, countries, institutions, journals, authors, references, and keywords.

**Results:**

A total of 751 articles were collected, the most active countries in this field were the United States and China. The authors with the most publications were Mackinnon, Susan E from the United States, and Xu WD and Gu YD from China. JOURNAL OF HAND SURGERY AMERICAN VOLUME was the journal with the most published. Based on keywords, the current research hotspots primarily revolved around nerve transfer, brachial plexus and reconstruction.

**Conclusion:**

The results of this bibliometric study provide clinical trends and hotspots in the surgical treatment of peripheral nerve injuries of the upper limb over the past 24 years, which may help researchers to identify clinical trends and explore new treatment in the field of peripheral nerve injuries.

## 1 Introduction

The peripheral nerves of the upper limb are governed by the brachial plexus (BP), which originates from the C5 to T1 segments of the spinal cord. Through complex branching and reorganization, the brachial plexus forms several major nerves that control the sensory and motor functions of the upper limb.

This includes patients with transections, lesions in continuity, entrapments, tumors, injection injuries, and birth palsies. Peripheral nerve injuries deemed to be Sunderland fourth-or fifth-degree injuries will require surgical intervention for recovery of function. Surgical techniques include nerve decompression, nerve suturing, nerve grafting, nerve transfer, and tendon transfer. Nerve decompression is used to relieve nerve entrapment, commonly applied in treating peripheral nerve compressions, while nerve suturing is used to directly repair transected nerves. Nerve grafting is employed to repair larger nerve defects by transplanting healthy nerve tissue to restore nerve function.

In cases of acute nerve injury caused by sharp objects, suturing is typically done within 72 h, allowing for end-to-end suturing. For blunt injuries, delayed suturing is performed after the formation of a neuroma at the end of nerve, which is then excised during surgery until normal nerve tissue is exposed and suturing can be performed. Spinner detailed the procedures for managing peripheral nerve injuries in his article (1).

Nerve grafting involves repairing damaged nerves by transplanting healthy nerve tissue, typically used when nerve defects are too large for direct suturing. This includes autologous nerve grafting, allogeneic nerve grafting, and synthetic nerve conduits. Autologous nerve grafting, the most common method, usually harvests the sural nerve from the patient’s own body. Allogeneic nerve grafting and synthetic nerve conduits are alternative options when autologous nerve resources are limited or unsuitable.

For severe and late-stage nerve injuries, surgical options primarily include nerve transfer and tendon transfer. Nerve transfer involves transferring a healthy donor nerve to the injured target nerve to restore its function. Nerve transfers can be motor or sensory, and well-established protocols currently exist for these procedures (2). This method is often used for severe nerve injuries, especially when the original nerve cannot be directly repaired or regenerated.

Over the past 20 years, several motor and sensory nerve transfers have been described in the upper limb, some are reliable and widely accepted. Nerve transfer is not just reserved for brachial plexus nerve root avulsions, it is being increasingly used to treat proximal peripheral nerve lesions. The determination of whether a peripheral nerve injury is reconstructed with nerve transfers and/or tendon transfers depends on several factors: the mechanism and location of injury, concomitant injuries and elapsed time from injury (3).

Surgical treatment of peripheral nerves had already been a research hotspot before 2000, with milestone surgical methods emerging. In 1903, Harris and Low stated end to side transfer of distal C5 root on the C6 root (4). The contralateral C7 root has been used by Gu since 1986 for complete C5-T1 avulsions of the brachial plexus, providing a new treatment option for brachial plexus root avulsion injuries (5). In 1994 Oberlin reported using a part of ulnar nerve for C5-C6 avulsion to restore elbow flexion (6). Thus named after Oberlin, this procedure became the classic surgical approach for functional reconstruction of the flexed elbow.

In view of the continued in-depth research in the field of surgical treatment of upper limb nerve injuries after 2000, this article uses bibliometric methods to query the literature in the field of peripheral nerve surgical treatment in the Web of Science database and utilizes the visualization capabilities of CiteSpace and VOSviewer software. The analysis aims to provide researchers with a reference for research trends and hotspots in this field.

## 2 Data and methods

### 2.1 Search strategy

The search utilized Web of Science to retrieve relevant literature, specifically leveraging the Web of Science Core Collection (WOSCC) database.^1^ The search strategy was TS = (“upper limb nerve” OR “musculocutaneous nerve” OR “median nerve” OR “ulnar nerve” OR “radial nerve” OR “axillary nerve” OR “suprascapular nerve” OR “subscapular nerve” OR “long thoracic nerve” OR “brachial plexus” OR “C5-T1”) AND TI = (“surgery” OR “surgical treatment” OR “nerve repair” OR “nerve grafting” OR “nerve transfer”) AND TS = (“human” OR “patients”) NOT TS = (“animal” OR “mice” OR “rat” OR “basic research” OR “*in vitro*” OR “complication” OR “anesthesia”).

The literature search was set from January 1, 2000, to June 14, 2024, yielding a total of 872 documents. Upon analyzing the retrieved literature, several issues were identified: (1) There were journal articles related to anesthesiology, mostly concerning nerve injuries caused during anesthesia procedures or discussing how to avoid surgical pain through anesthesia. (2) Some literature described nerve injuries or complications caused by surgery. This was not aligned with our required content, which focused on the surgical treatment of upper limb peripheral nerve injuries. Therefore, we needed to exclude irrelevant search content.

The first refinement was done by LANGUAGES: (ENGLISH); NOT research areas: Anesthesiology, resulting in 764 documents. The second refinement was by DOCUMENT TYPES: (ARTICLE OR REVIEW PAPER), yielding a total of 751 documents. Below is my search process and refinement steps (Table 1). The retrieved content included article titles, authors, abstracts, keywords, and all citations, which were downloaded in plain text format. The search was concluded on June 14, 2024.

**Table 1:** Search strategy.

Set	Number of results	Refinement
1	872	TS = (“upper limb nerve” OR “musculocutaneous nerve” OR “median nerve” OR “ulnar nerve” OR “radial nerve” OR “axillary nerve” OR “suprascapular nerve” OR “subscapular nerve” OR “long thoracic nerve” OR “brachial plexus” OR “C5-T1”) AND TI = (“surgery” OR “surgical treatment” OR “nerve repair” OR “nerve grafting” OR “nerve transfer”) AND TS = (“human” OR “patients”) NOT TS = (“animal” OR “mice” OR “rat” OR “basic research” OR “*in vitro*” OR “complication” OR “anesthesia”)
2	764	Refined by LANGUAGES: (ENGLISH); NOT research areas: Anesthesiology
3	751	Refined by DOCUMENT TYPES: (ARTICLE OR REVIEW PAPER)

### 2.2 Data extraction and bibliometric analysis

The retrieved literature was first subjected to preliminary analysis using the Analyze Results and Citation Report functions of Web of Science. Subsequently, the collected bibliographic data were processed using the bibliometric software VOSviewer (Ver. 1.6.20) and CiteSpace (Ver. 6.3.R1 advanced). The key steps were as follows:

1. Export all records of the search results, including author, title, source, and abstract. Use CiteSpace software to analyze the data in plain text file format and VOSviewer software to analyze the data in tab-delimited file format. Node types were selected for country, institution, author, keyword, cited journal, cited author, and cited reference. Time Slicing was set from January 1, 2000, to June 14, 2024, with the time partitioned to 1 year, and the number of Years Per Slice (1). Each node in the map represents an element, such as an author, keyword, institution, etc.
2. Create a Keyword co-occurrence plots in VOSviewer to identify frequently occurring keywords and generate a keyword co-occurrence network map and key word density visualization.
3. Use CiteSpace software to perform keyword clustering analysis, keyword burst analysis, and reference co-citation Analysis of the literature. This helps identify key literature and citation bursts in the research field, revealing the flow of knowledge and development trends within the domain.
4. Interpret and discuss the charts generated by VOSviewer and CiteSpace based on the analyses.

## 3 Results

### 3.1 Global publishing trends

#### 3.1.1 Annual publication volume

**Figure 1:**
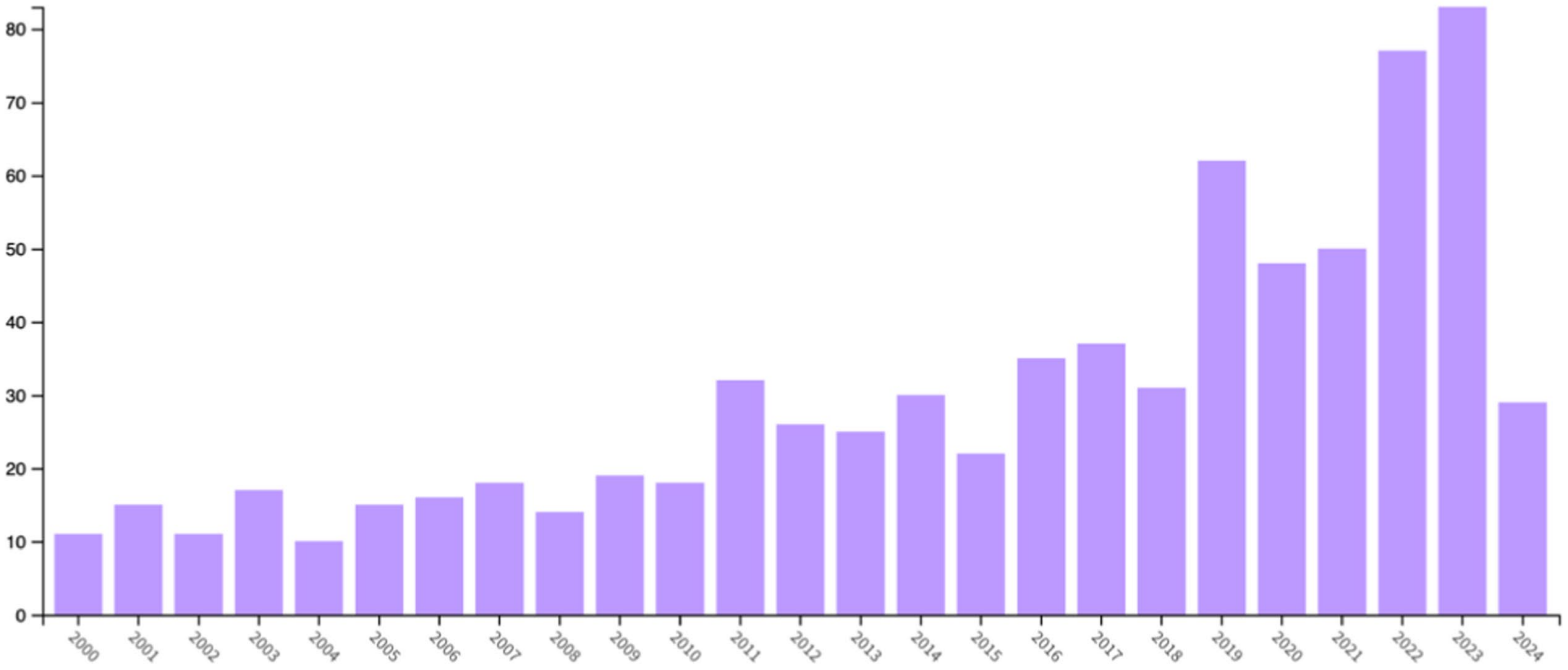
Annual publication volume from 2000 to 2024.

The exported literature results were analyzed in Web of Science, yielding a total of 751 documents. The results are presented in the form of a bar chart (Figure 1), showing a steady upward trend in the number of publications over the 24 years. The number of publications remained stable from 2000 to 2010, showed a slow growth trend from 2011 to 2018, and experienced significant growth in 2019 (62 articles). There was a substantial increase in 2022 (77 articles, 10.253%) and 2023 (83 articles, 11.052%).

#### 3.1.2 Countries

**Figure 2:**
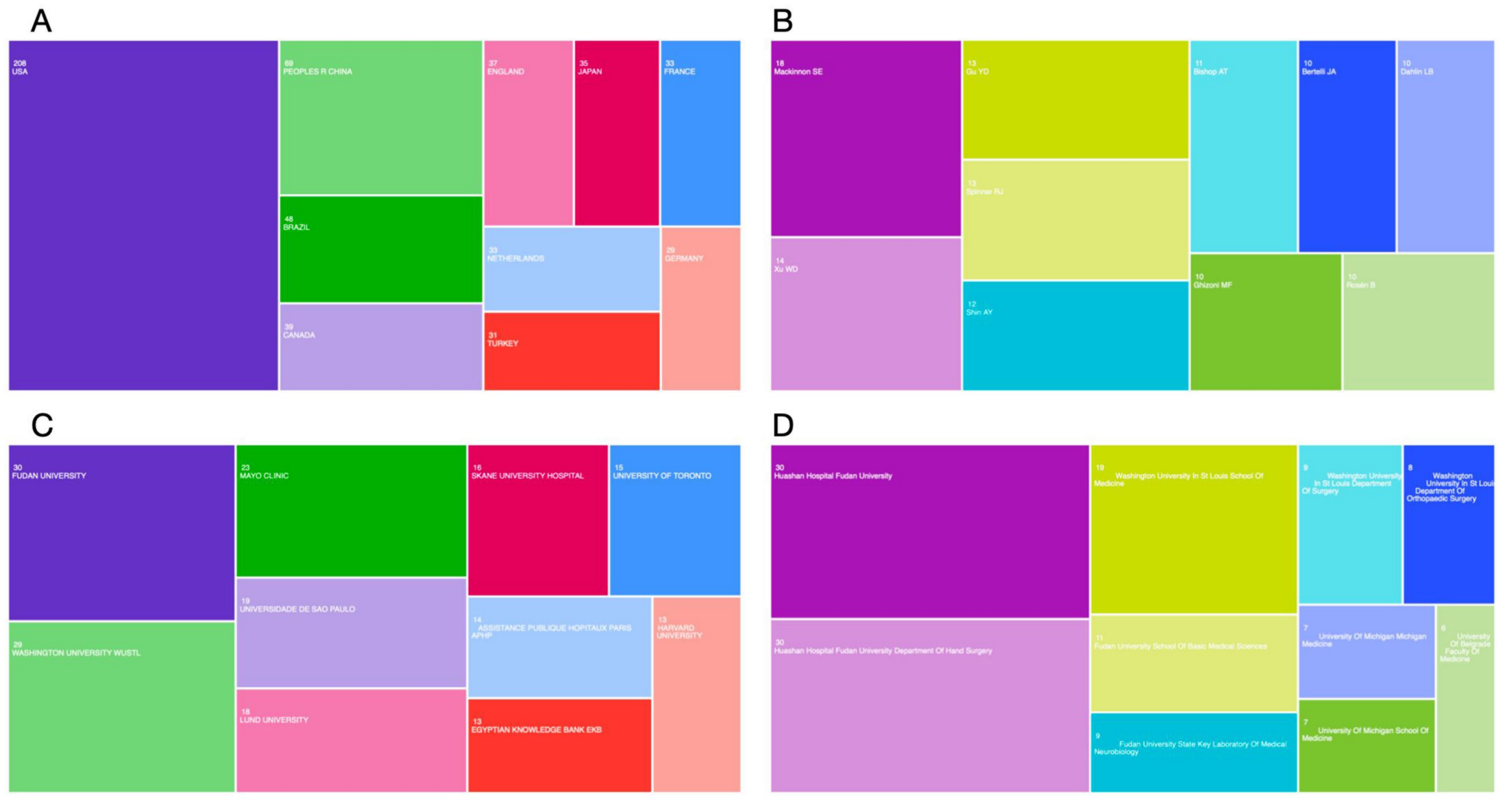
Global publishing in the surgical treatment of peripheral nerves of the upper limb. **(A)** Volume of publications by country and region from 2000 to 2024. **(B)** Volume of publications by author. **(C)** Volume of publications by affiliation. **(D)** Volume of publications by affiliation with department.

The exported literature results were analyzed in Web of Science and presented in a Tree Map Chart (Figure 2A). Among the countries where the literature was published, the United States ranked first with 208 articles, followed by China with 69 articles, and Brazil with 48 articles. Other countries included Canada (39 articles), the United Kingdom (37 articles), Japan (35 articles), France (33 articles), the Netherlands (33 articles), Turkey (31 articles), and Germany (29 articles).

#### 3.1.3 Authors

The exported literature results were analyzed in Web of Science and presented in a TreeMap Chart (Figure 2B). From 2020 to 2024, Mackinnon, Susan E from the United States published the most articles (18 articles), followed by Xu WD from China (14 articles) and Gu YD from China (13 articles).

#### 3.1.4 Affiliation; affiliation with department

The exported literature results were analyzed in Web of Science and presented in a TreeMap Chart (Figures 2C,D). From an institutional perspective, Fudan University published the most content on the surgical treatment of peripheral nerve injuries (30 articles), followed by Washington University (29 articles) and Mayo Clinic (23 articles). From the perspective of institutions and departments, the Huashan Hospital Fudan University and the Huashan Hospital Fudan University department of hand surgery published the most (30 articles each), with Washington University in St. Louis School of Medicine ranking third (19 articles).

#### 3.1.5 Published journals

The journal with the most publications between 2000 and 2024 was the Journal of Hand Surgery American Volume, with 52 articles (6.924%), followed by the Journal of Neurosurgery with 31 articles (4.128%). The journals covered a range of JCR categories from Q1 to Q4. Notably, the highest impact factor (IF) among the published articles was 96.2. This highest IF article, published in the NEW ENGLAND JOURNAL OF MEDICINE, was titled “Trial of Contralateral Seventh Cervical Nerve Transfer for Spastic Arm Paralysis.” The Table 2 lists the top 10 journals by the number of publications.

**Table 2:** TOP 10 journals.

Rank	Journal	Count	Percentage (%)	IF (2023)	JCR
1	Journal of Hand Surgery American Volume	52	24.07	2.1	Q2
2	Journal of Neurosurgery	31	14.35	3.5	Q1
3	Plastic and Reconstructive Surgery	26	12.03	3.2	Q1
4	Microsurgery	24	11.11	1.5	Q3
5	Journal of Hand Surgery European Volume	16	7.41	1.892	Q3
6	Neurosurgery	16	7.41	3.9	Q1
7	Hand Surgery Rehabilitation	14	6.48	0.9	Q4
8	Hand American Association for Hand Surgery	13	6.02	1.8	Q2
9	Journal of Plastic Reconstructive and Aesthetic Surgery	13	6.02	2	Q2
10	Acta Neurochirurgica	11	5.09	1.9	Q3

Top 10 journals by the number of publications.

### 3.2 Keyword analysis

Keyword analysis through VOSviewer and CiteSpace software can display the keywords with the highest frequency and the specific time when keywords appear together and can also reflect changes in research hotspots.

**Figure 3:**
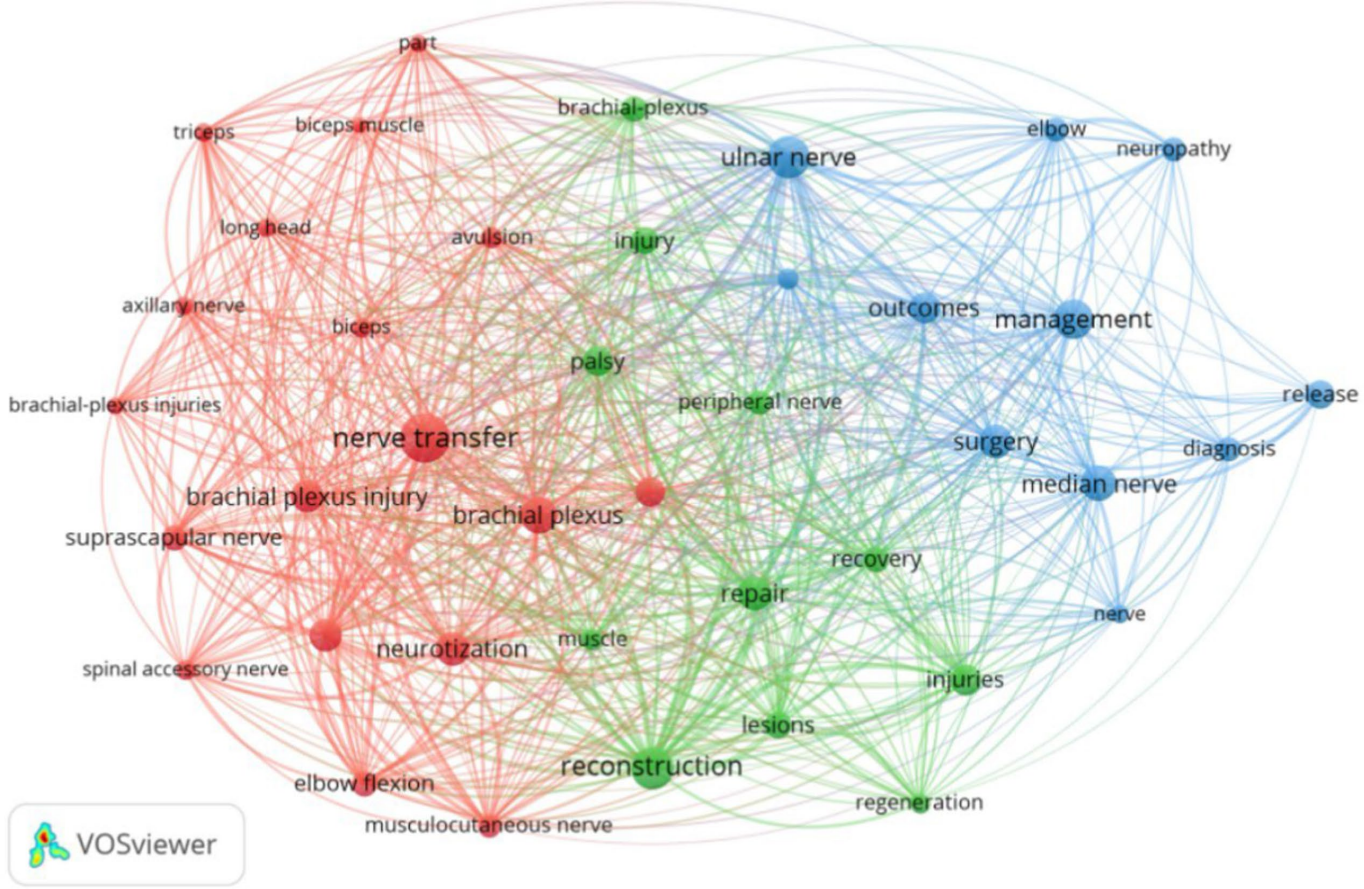
Key word network visualization where the 40 keywords are divided into 3 clusters. The lines between the nodes (keywords) represent co-occurrence relationships. The colors (red, green, and blue) represent clusters of keywords that frequently co-occur. Larger nodes indicate keywords that appear more frequently in the dataset.

**Figure 4:**
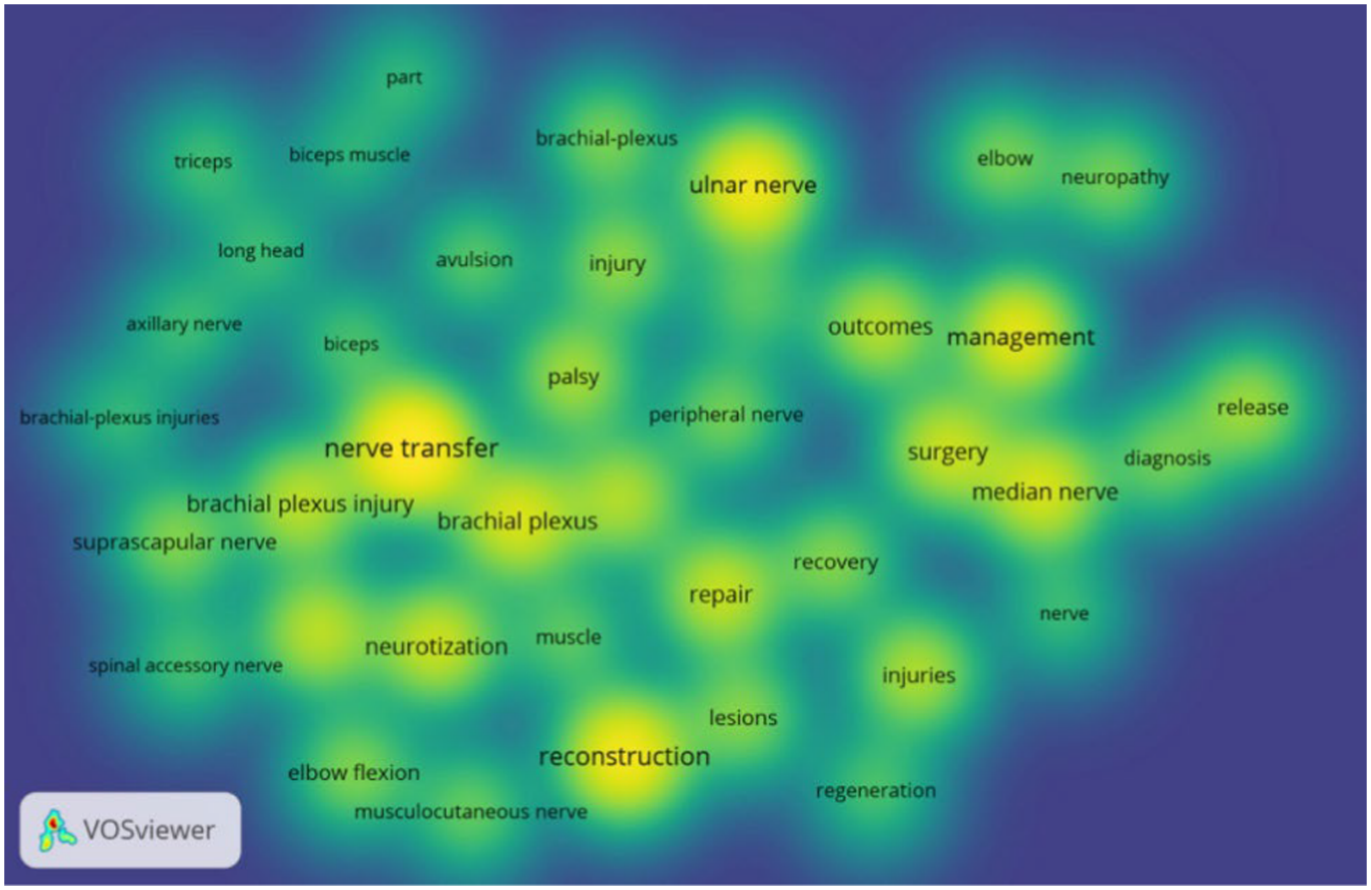
Keyword density map, where larger kernel indicates a higher frequency of appearance.

Keyword co-occurrence plots were obtained from keyword analysis in the literature using VOSviewer software. Figure 3 shows keyword network visualization, where the 40 keywords are divided into 3 clusters. Cluster 1 contains 18 keywords, including avulsion, axillary nerve, biceps, biceps muscle, brachial plexus, brachial plexus injury, brachial plexus injuries, elbow flexion, long head, musculocutaneous nerve, nerve transfer, neurotization, part, restoration, shoulder, spinal accessory nerve, suprascapular nerve, and triceps. Cluster 2 includes 11 keywords, such as brachial plexus, injuries, injury, lesions, muscle, palsy, peripheral nerve, reconstruction, recovery, regeneration, and repair. Cluster 3 includes 11 keywords, including diagnosis, elbow, management, median nerve, nerve, neuropathy, outcomes, release, surgery, surgical treatment, and ulnar nerve. Each node represents a keyword, and the size of the node represents the frequency of the keyword. Based on frequency, we found the top 10 keywords: nerve transfer, brachial plexus, reconstruction, injury, ulnar nerve, brachial plexus injury, management, median nerve, repair, and restoration (Table 3). This Figure 4 shows the keyword density visualization; each kernel shows the item density. These keywords highlight the hotspots in surgical treatment for upper limb peripheral nerve injuries from 2000 to 2024.

**Table 3:** TOP-10 frequency of key word.

Rank	Frequency	Year	Key word
1	131	2001	Nerve transfer
2	112	2000	Brachial plexus
3	104	2003	Reconstruction
4	100	2003	Injury
5	97	2003	Ulnar nerve
6	93	2000	Brachial plexus injury
7	84	2001	Management
8	77	2002	Median nerve
9	59	2003	Repair
10	56	2002	Restoration

**Figure 5:**
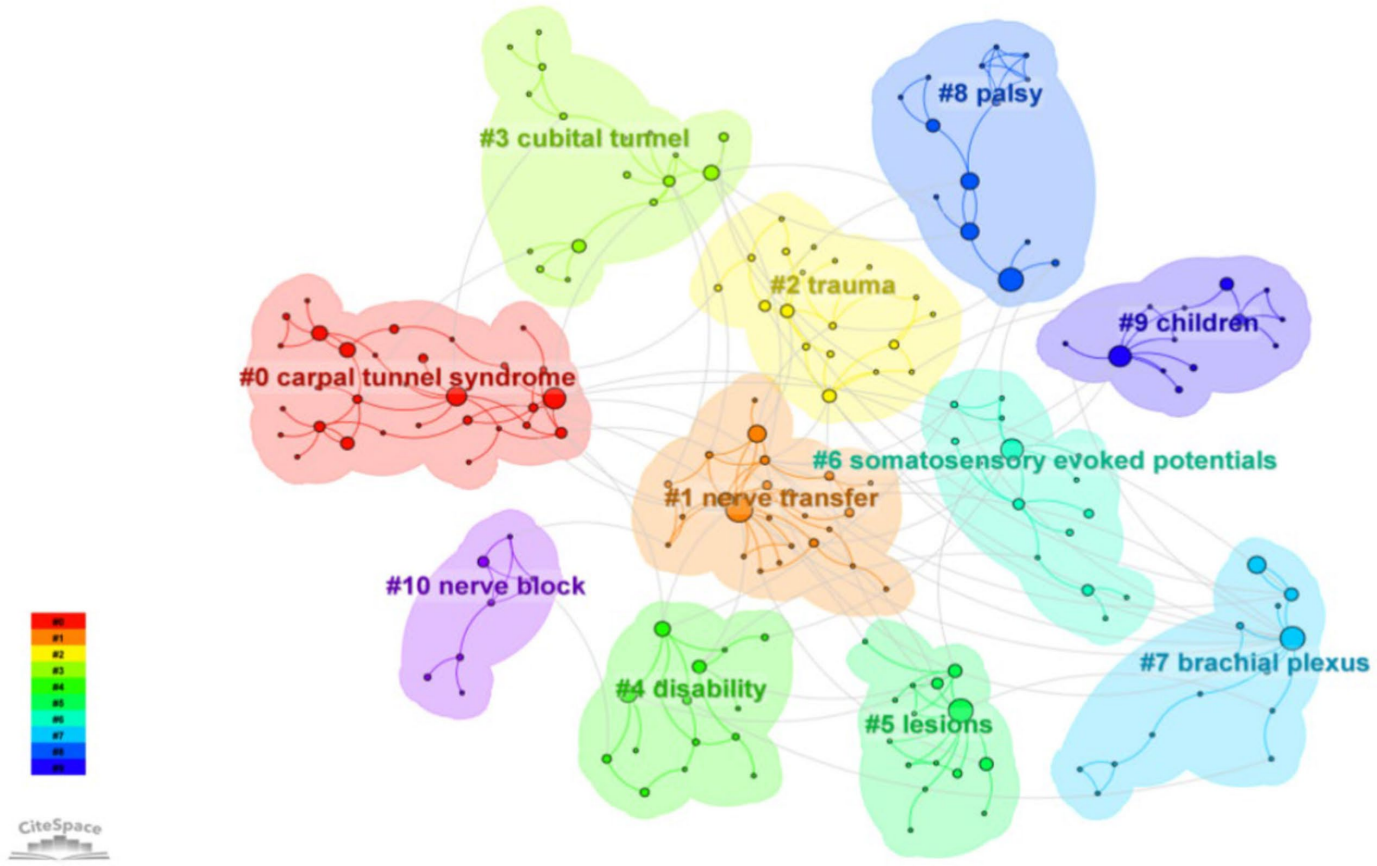
Keyword clustering analysis. Each color corresponds to a cluster. The keywords were clustered into 10 labels. The labels are derived from the most representative keywords or terms within each cluster. Larger nodes represent keywords that appear more frequently in the dataset.

The keywords were also analyzed by clustering using CiteSpace software and the keywords were clustered into 10 labels with the clustering labels derived from the LLR algorithm (Figure 5). The 10 clustering labels are: carpal tunnel syndrome nerve transfer trauma cubital tunnel disability lesions somatosensory evoked potentials brachial plexus palsy children and nerve block. “Carpal tunnel syndrome” (red cluster) and “nerve transfer” (orange cluster) have larger nodes indicating they are central and frequently occurring terms in this research area. This table shows the specific clustering information (Table 4). These clustered keywords can reflect the research hotspots in surgical treatment of upper limb nerve injuries

**Table 4:** Keyword clustering labels for research areas on surgical treatment of peripheral nerve injuries of the upper limb from 2000 to 2024.

Cluster ID	Size	Silhouette	Mean (year)	Label (LLR)
0	30	0.964	2012	Carpal tunnel syndrome (62, 1.0E-4)
1	24	0.79	2010	Nerve transfer (26.68, 1.0E-4)
2	20	0.917	2015	Trauma (9.32, 0.005)
3	17	0.876	2013	Cubital tunnel (31.9, 1.0E-4)
4	15	0.741	2009	Disability (19.16, 1.0E-4)
5	15	0.908	2008	Lesions (9.42, 0.005)
6	15	0.844	2009	Somatosensory evoked potentials (11.03, 0.001)
7	14	0.953	2007	Brachial plexus (26.37, 1.0E-4)
8	14	0.929	2010	Palsy (12.53, 0.001)
9	13	0.92	2007	Children (20.82, 1.0E-4)
10	7	1	2019	Nerve block (16.65, 1.0E-4)

**Figure 6:**
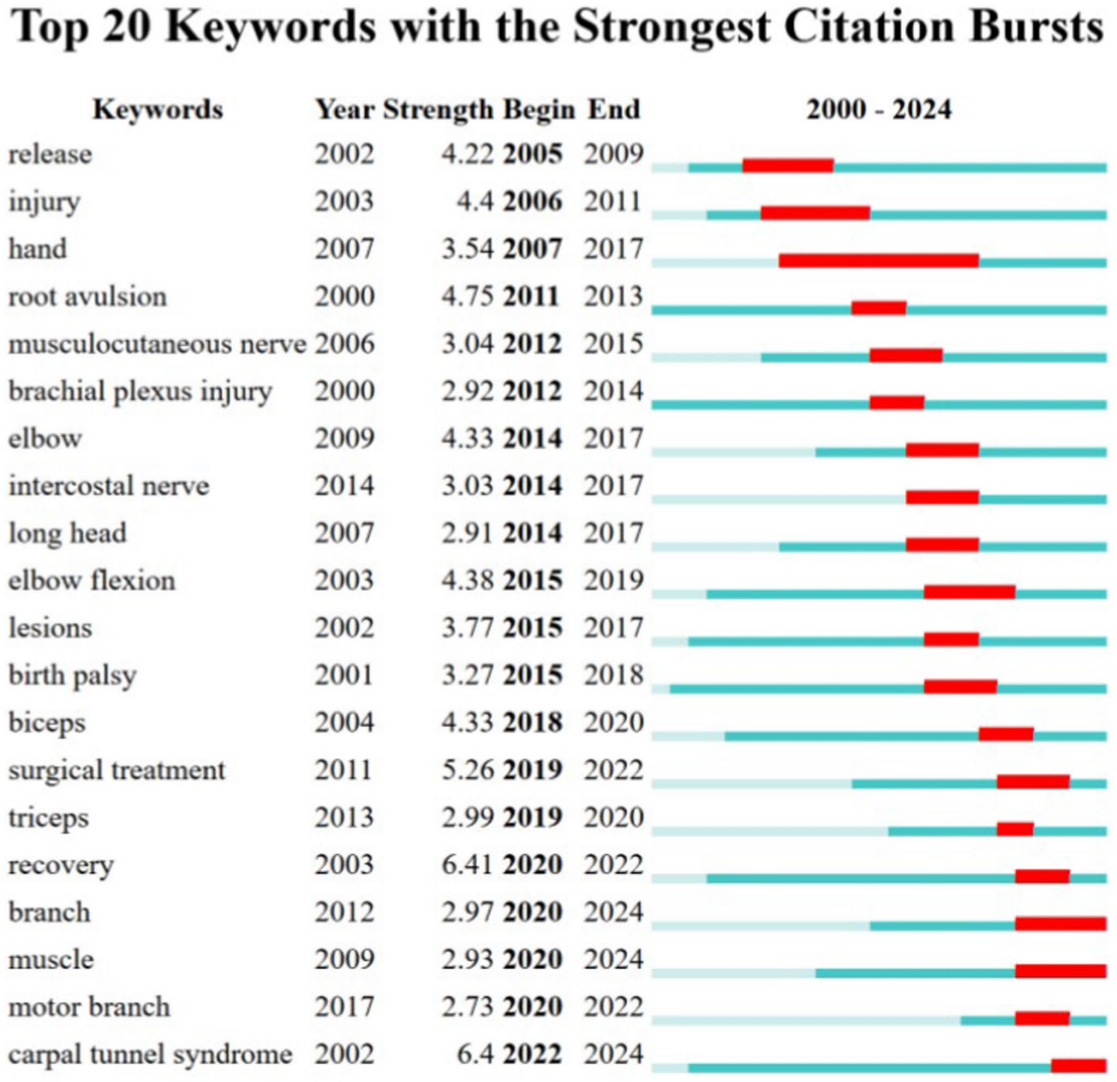
Top 20 keywords with the strongest citation bursts.

CiteSpace was also utilized to detect keyword bursts. Figure 6 displays the top 20 keywords with the highest burst strength. The earliest keyword burst was “release,” the longest-lasting burst was “hand,” and the most recent burst was “carpal tunnel syndrome.” Through keyword analysis, we can identify the recent hotspots in upper limb nerve research.

### 3.3 Reference co-citation analysis

**Figure 7:**
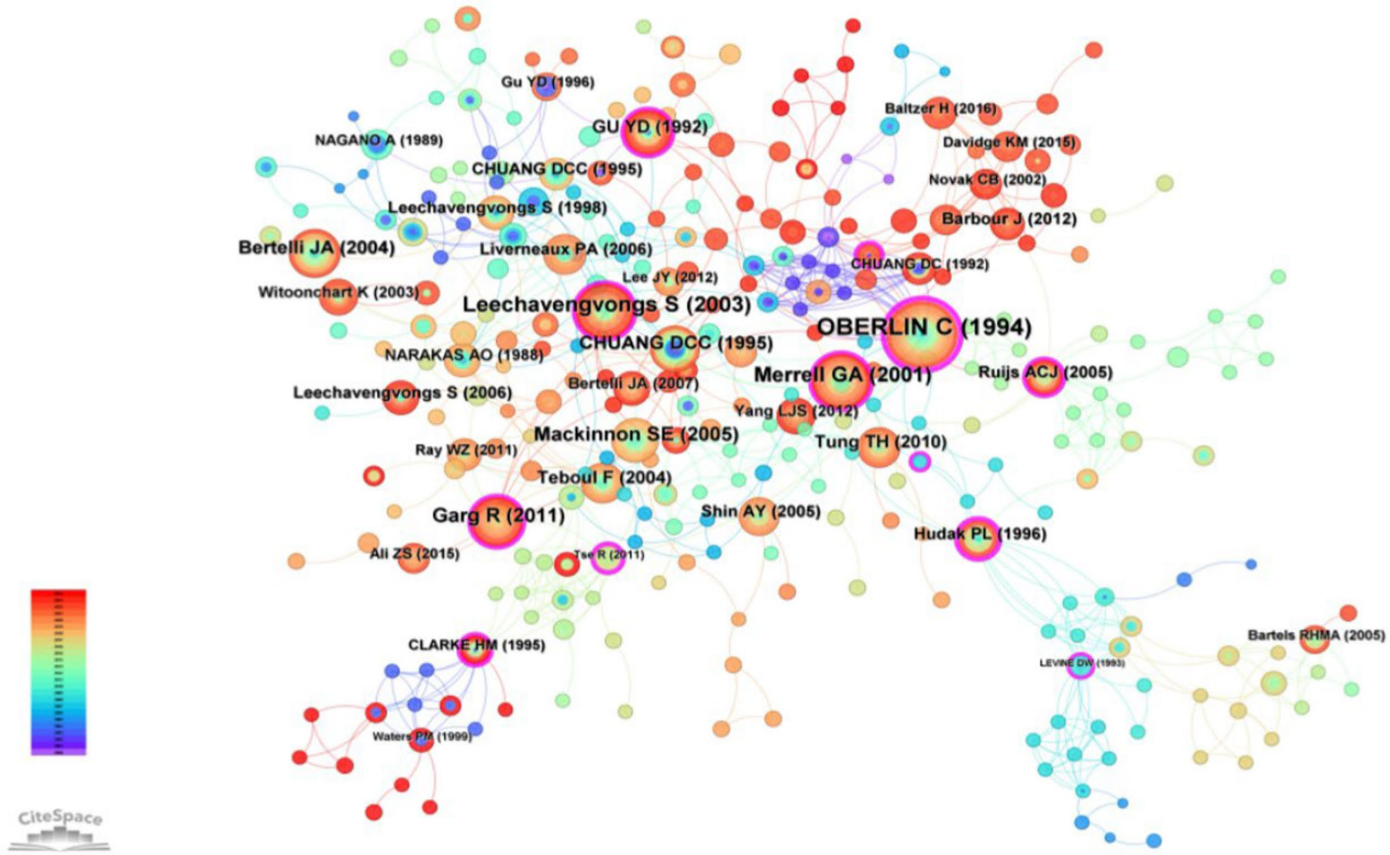
Reference co-occurrence network in the surgical treatment of upper limb nerve. The colors of the nodes represent the time of publication of the references, as indicated by the color gradient legend on the left, red node means the most recent references in the network. Larger nodes indicate references with a higher number of citations or co-citations in the network, these are key references or highly influential works in the field. Nodes with colored rings around them indicate citation bursts. Nodes like Oberlin et al. (6), Leechavengvongs et al. (8), and Mackinnon et al. (33) are central and well-connected. These are seminal and influential works frequently cited in the context of this field.

The most frequently co-cited articles in the field of surgical treatment of upper limb nerve research were compiled and listed by CiteSpace software to obtain co-citation and highlighting plots of the literature (Figure 7). In the figure, the larger the diameter of the node, the higher the co-citation frequency. The most frequently cited article was cited 71 times, which is the 1994 paper by Oberlin C titled Nerve transfer to biceps muscle using a part of ulnar nerve for C5-C6 avulsion of the brachial plexus: anatomical study and report of four cases. The following table (Table 5) lists the top 10 most frequently co-cited articles, all of which are milestone papers in the surgical treatment of upper limb peripheral nerve injuries and have made pioneering contributions in this field.

**Table 5:** Top 10 most frequently co-cited articles.

Rank	Count	Centrality	Year	References
1	71	0.42	1994	OBERLIN C, 1994, J HAND SURG-AM, V19A, P232, DOI 10.1016/0363-5023(94)90011-6
2	45	0.15	2003	Leechavengvongs S, 2003, J HAND SURG-AM, V28A, P633, DOI 10.1016/S0363-5023(03)00199-0
3	40	0.18	2001	Merrell GA, 2001, J HAND SURG-AM, V26A, P303, DOI 10.1053/jhsu.2001.21518
4	32	0.16	2011	Garg R, 2011, J BONE JOINT SURG AM, V93A, P819, DOI 10.2106/JBJS.I.01602
5	29	0.09	2005	Mackinnon SE, 2005, J HAND SURG-AM, V30A, P978, DOI 10.1016/j.jhsa.2005.05.014
6	24	0.01	2004	Bertelli JA, 2004, J HAND SURG-AM, V29A, P131, DOI 10.1016/j.jhsa.2003.10.013
7	23	0.08	1995	CHUANG DCC, 1995, PLAST RECONSTR SURG, V96, P122, DOI 10.1097/00006534-199507000-00019
8	22	0.1	1992	GU YD, 1992, J HAND SURG-BRIT EUR, V17B, P518, DOI 10.1016/S0266-7681(05)80235-9
9	20	0.09	2004	Teboul F, 2004, J BONE JOINT SURG AM, V86A, P1485, DOI 10.2106/00004623-200407000-00018
10	20	0.07	2010	Tung TH, 2010, J HAND SURG-AM, V35A, P332, DOI 10.1016/j.jhsa.2009.12.002

Upon reviewing the content of the top 10 articles, we found that except for the 4th and 10th articles, the other papers described surgical treatments for shoulder and elbow function reconstruction following brachial plexus injury, primarily focusing on the reconstruction of shoulder abduction and elbow flexion functions. This highlights that “nerve transfer,” “brachial plexus injury,” and “reconstruction” have been the research hotspots and frontier issues for surgeons over the past 24 years. Upper limb peripheral nerve injuries, particularly brachial plexus injuries, result in loss of limb motor and sensory functions. The main goal of surgery is to restore limb function, especially motor function. In 1992, Gu proposed an innovative technique for treating brachial plexus root avulsion injuries by transferring the seventh cervical nerve root from the contralateral healthy side (5). This operation offers a new approach for the treatment of brachial plexus root avulsion. This operation offers a new approach for the treatment of brachial plexus root avulsion. Research has found that transferring the healthy side’s seventh cervical nerve to the paralyzed side’s seventh cervical nerve can significantly improve the strength and function of the paralyzed arm and reduce spasms, providing a new treatment option for brachial plexus root avulsion injuries. This innovative achievement shifted the focus of brachial plexus treatment from peripheral nerve injury to central nerve injury, thereby opening a new field of “treating central nerve injuries through peripheral function changes.” In the 1994 article by Oberlin, the author described the use of 10% of the bulk of the ulnar nerve to directly suture the motor nerve of the biceps muscle to reconstruct elbow flexion function in patients with C5-C6 brachial plexus avulsion injuries, without significantly impairment of hand function. This technique was later named after Oberlin himself and became the most representative surgery for elbow flexion reconstruction (6). In 1995 Chuang reported the effect of spinal accessory nerve transfer to the suprascapular nerve for shoulder abduction function reconstruction. This article laid the foundation for future shoulder abduction function reconstruction (7). In 2003 Leechavengvongs reports the results of nerve transfer to the deltoid muscle using the nerve to the long head of the triceps. This method is a reliable and effective procedure for deltoid reconstruction in brachial plexus injury (upper-arm type) and combined with spinal accessory nerve transfer to the suprascapular nerve to obtain good shoulder abduction, providing a relatively fixed choice for shoulder abduction function reconstruction (8).

## 4 Discussion

Based on the analysis results from CiteSpace and VOSviewer, “nerve transfer,” “brachial plexus injury,” and “reconstruction” have been the most popular and highly regarded topics over the past 24 years. This is mainly due to the functional impairments caused by brachial plexus injuries, particularly severe proximal brachial plexus injuries, which result in significant functional disabilities and urgently require solutions. Moreover, brachial plexus injuries are among the most challenging upper limb peripheral nerve injuries to treat, with the most diverse surgical approaches. In general, the optimal time of surgical intervention with nerve transfers is before 6 months. Early exploration and reconstruction with nerve transfers between 3 and 6 weeks is indicated when there is a high suspicion of root avulsion or a proximal nerve injury that will not reach the distal motor endplate. Routine exploration is performed 3–6 months after injury in patients who have not demonstrated adequate reinnervation. Results from delayed (9–12 months) or late (>12 months) nerve transfers are poor. It is in these cases that tendon transfer or a microvascular transfer of a normal muscle in conjunction with an extraplexus motor nerve transfer is recommended (3).

### 4.1 Surgical treatment of upper-arm type brachial plexus injury

Upper-arm type brachial plexus injuries typically affect the C5 and C6 nerve roots, leading to dysfunction in shoulder abduction and external rotation, as well as elbow flexion. These injuries usually require surgical intervention. Functionally, restoring elbow flexion is the highest priority, followed by restoring shoulder abduction, shoulder stability, and shoulder external rotation. The primary surgical methods are nerve transfer and tendon transfer. In cases of severe proximal brachial plexus injuries, nerve transfer is the only option for reconstructing upper limb function.

#### 4.1.1 Nerve transfer

Regarding elbow flexion reconstruction, the classic 1994 article by Oberlin described the use of a portion of the ulnar nerve fascicles transferred to the motor nerve of the biceps muscle, which became a representative technique for elbow flexion reconstruction (6). In 2001, Merrell reported Better results for restoration of elbow flexion have been attained with intercostal to musculocutaneous transfers than with spinal accessory nerve transfers and spinal accessory to suprascapular transfers appear to have the best outcomes for return of shoulder abduction (9). Garg stated, “In patients with demonstrated complete traumatic upper brachial plexus injuries of C5-C6, the pooled international data strongly favors dual nerve transfer over traditional nerve grafting for restoration of improved shoulder and elbow function.” The advantage of dual nerve transfer lies in its ability to provide stronger nerve supply, thereby enhancing muscle function recovery. Additionally, dual nerve transfer can reduce the length of nerve grafts required, lowering the complexity of the surgery and the risk of complications (10).

In 2003, Witoonchart experimentally evaluated the feasibility of restoring the motor function of the deltoid muscle with complete C5-C6 root injury by transferring the nerve to the long head of the triceps to the anterior branch of the axillary nerve through a posterior approach (11). Subsequent studies by Leechavengvongs reported the results of nerve transfer to the deltoid muscle using the nerve to the long head of the triceps. This method is a reliable and effective procedure for deltoid reconstruction in upper-arm type brachial plexus injury. Combined with spinal accessory nerve transfer to the suprascapular nerve, it provides a consistent choice for shoulder abduction reconstruction (8). Kim suggested that, if patients are thoroughly evaluated, surgical exploration and repair of brachial plexus lesions is technically feasible and favorable outcomes can be achieved. Repairs were best for injuries located at the C-5, C-6, and C-7 levels, the upper and middle trunk, the lateral cord to the musculocutaneous nerve, and the median and posterior cords to the axillary and radial nerves (12).

Following numerous publications on nerve transfer surgical techniques, researchers began to examine the outcomes of nerve transfer versus nerve repair. Treatment outcomes vary depending on the nature of the nerve injury. The 2012 study by Yang found results consistent with those of Garg and colleagues, indicating that nerve transfer is more effective than nerve repair in restoring elbow flexion. For isolated loss of elbow flexion, nerve transfer is the ideal strategy. However, the study did not show significant differences in shoulder abduction recovery between nerve repair, nerve transfer, and nerve transfer combined with proximal nerve repair. It does not support the use of nerve transfer alone to treat upper brachial plexus injuries without understanding the pathological anatomy necessary for optimizing nerve reconstruction strategies (13). Forli recommend for complete brachial plexus injuries, if one or two roots are usable, nerve grafting of one or two roots is recommended and can be combined with nerve transfer for the Suprascapular nerve and on the motor branch of the Triceps brachii muscle. If no root can be grafted, it is recommended at a minimum to transfer Intercostal nerves to restore elbow flexion with external branch of the accessory nerve transfer on the Suprascapular nerve (14).

For partial lesions at C5, C6, C7, an intraplexus donor nerve has better results. Elbow flexion is ensured by single transfer (Oberlin’s technique) or double transfer. Shoulder function is ensured by transferring a motor branch of the triceps brachii muscle on the axillary nerve or external branch of the accessory nerve transfer onto the suprascapular nerve. Bertelli and Ghizoni report the best results in terms of restoring shoulder and elbow function when nerve transfer is combined with a nerve graft of the C5 ± C6 roots. When one of these roots is usable, they recommend supplementing the nerve transfer (EBAN onto SS + ulnar onto BB + TB onto axillary) by a nerve graft of the superior roots (C5 ± C6) on the anterior and posterior divisions, respectively, of the superior trunk (15).

Another important nerve transfer method is the contralateral C7 nerve root transfer. The C7 nerve root is centrally located in the brachial plexus and independently forms the middle trunk. The primary muscles of the upper limb are not solely innervated by C7 and can be compensated by other nerve roots. Therefore, severing the contralateral C7 nerve root does not result in permanent functional loss of the non-affected upper limb (16). In 1992, Gu utilized contralateral C7 transfer to treat brachial plexus root avulsion injuries, which can reconstruct the function of the upper arm and forearm. Postoperative recovery of elbow flexion was good, and wrist flexion and finger flexion could recover to M3/M4 (5). In 2018, Huashan Hospital investigated the effect of grafting the contralateral C7 nerve from the nonparalyzed side to the paralyzed side in patients with spastic arm paralysis, transfer of the C7 nerve from the nonparalyzed side to the paralyzed side associated with a greater improvement in function and reduction of spasticity than rehabilitation alone over a period of 12 months (17). The contralateral C7 nerve transfer surgery has shown significant clinical efficacy in treating spastic arm paralysis, providing a new treatment option for this patient population.

#### 4.1.2 Tendon transfer

Shoulder external rotation should take priority because this function will allow patients to position their hand in front of their body. With a functional elbow and hand, patients will be able to do most activities of daily living. The lower trapezius can be a good transfer to restore external rotation of the shoulder. Other parts of the trapezius, levator scapulae, rhomboids, and, when available, the latissimus dorsi, pectoralis major, teres major, biceps, triceps, and serratus anterior muscles can all be used to replace the rotator cuff and deltoid muscle function (18). Lower trapezius potentially results in superior restoration of shoulder external rotation with the arm at the side compared with latissimus dorsi and should be considered as a potential tendon transfer to restore external rotation (19).

### 4.2 Surgical treatment of lower trunk brachial plexus injury

Lower trunk type injuries involve the C8-T1 segments of the brachial plexus and lead to dysfunction of the wrist and hand. Because of the great distance between the proximal plexus and the neuromuscular endplate, restoration of hand function are poor compared to the shoulder or the elbow. Therefore, tendon and muscle transfers are frequently used to restore hand function.

For reconstructing wrist and hand function, many classic tendon transfer techniques are already established (20, 21). Tendon transfers are advantageous in that they result in return of function soon after the immobilization period (within 4–6 weeks). However, nerve transfers are also important because they restore more natural motor function and avoid sacrificing donor muscles.

During the past decade the central nervous system’s ability to register effective function to even the most creative nerve transfers has been demonstrated (22). An understanding of the redundancy of innervation to particular muscle groups or movements has enabled the peripheral nerve surgeon to identify a number of options for donor nerves or fascicles within the forearm (23).

Ulnar nerve injuries often lead to the loss of intrinsic hand muscle function, affecting fine motor skills and grip strength. Repair and reconstruction of intrinsic hand muscle function represent a significant challenge in peripheral nerve injury. Novak and Mackinnon explored the surgical method of transferring the distal branch of the anterior interosseous nerve (AIN) to the pronator quadratus to the deep branch of the ulnar nerve. By direct nerve coaptation, the regeneration distance is shortened, and the regeneration effect is improved (24). All patients had reinnervation of the ulnar nerve intrinsic muscles of the hand. By transferring the AIN to the deep motor branch of the ulnar nerve, these muscles can be reinnervated, restoring partial intrinsic hand muscle function. This study provided a new surgical approach for the recovery of intrinsic hand muscle function. In 2012, Barbour, building on previous research, proposed supercharged end-to-side (SETS) nerve transfer technique instead of end-to-end nerve transfer. He described the technique for a SETS nerve transfer of the terminal anterior interosseous nerve to the pronator quadratus muscle end-to-side to the deep motor fascicle of the ulnar nerve in the distal forearm. The author believed that the SETS procedure might have broad clinical utility for second-and third-degree axonotmetic nerve injuries, to augment partial recovery and/or “babysit” motor end plates until the native parent axons regenerate to target (25).

### 4.3 Entrapment neuropathies of the upper extremity

Entrapment neuropathies constitute a significant category of peripheral nerve injuries in the upper extremity. They are well-recognized conditions with a high incidence rate. Over years of clinical and research efforts, there are now well-established treatment protocols with confirmed efficacy for these conditions. As illustrated in Figure 6, recent years have seen a renewed interest in entrapment neuropathies, particularly carpal tunnel syndrome (CTS). The current research focus is on diagnostic imaging techniques and fundamental studies of entrapment neuropathies.

The main types of entrapment neuropathies include median nerve entrapment, ulnar nerve entrapment, radial nerve entrapment, and thoracic outlet syndrome. Median nerve entrapment at the wrist, which manifests as carpal tunnel syndrome, is the most common form. This is followed by ulnar nerve entrapment at the elbow, known as cubital tunnel syndrome. Carpal tunnel syndrome and cubital tunnel syndrome are the most prevalent types of entrapment neuropathies. Thoracic outlet syndrome is increasingly recognized as a distinct clinical entity. The primary surgical intervention for entrapment neuropathies is nerve decompression, a topic that has been extensively studied with numerous related publications. Most of these publications focus on the treatment of carpal tunnel syndrome (Figure 5). Studies have shown that surgical intervention for carpal tunnel syndrome is more effective than cast immobilization (26). Randomized controlled trials (RCTs) have demonstrated that surgery significantly alleviates symptoms compared to corticosteroid injections (27). Surgical treatments for carpal tunnel syndrome include open carpal tunnel release (OCTR) and endoscopic carpal tunnel release (ECTR). There is substantial research comparing the efficacy of open versus endoscopic decompression of the median nerve. Li et al. concluded that, overall, evidence from randomized controlled trials indicates that ECTR results in better recovery of daily life functions compared to OCTR. This is evidenced by higher satisfaction rates, greater key pinch strengths, earlier return to work times, and fewer scar-related complications. This findings suggest that patients with CTS can be effectively managed with ECTR (28). As depicted in the chart (Fig: keyword bursts), carpal tunnel syndrome has re-emerged as a research hotspot in the past 2 years. Current interest in carpal tunnel syndrome includes the use of ultrasound for diagnosis (29) and the study of MRI criteria for diagnosing and predicting severity of carpal tunnel syndrome (30). These imaging modalities offer non-invasive advantages over traditional electromyography. Furthermore, ultrasound can be utilized not only for diagnostic purposes but also for therapeutic interventions under ultrasound guidance. Ultrasound-guided carpal tunnel release represents a significant advancement in CTS treatment, offering a minimally invasive option with excellent outcomes. Meanwhile, research continues to expand in areas such as advanced imaging, regenerative therapies, and wearable technology, making CTS a vibrant area of clinical and scientific interest. These innovations offer new hope for patients and are shaping the future of CTS diagnosis, treatment, and prevention. Recent evidence indicates that the prevalence of carpal tunnel syndrome has increased in the elderly population over 75 years of age. Additionally, some rare systemic diseases, such as amyloidosis, may present early manifestations as carpal tunnel syndrome. This underscores the importance for clinicians to be vigilant about these rare diseases, as early diagnosis and treatment are critical for improving patient outcomes (31). Moreover, Rydberg et al. found that CTS could be early indicators of preclinical type 2 diabetes (T2D). Early detection of T2D is crucial to prevent associated complications and morbidity (32).

### 4.4 Prospective analysis of research on surgical treatment of peripheral nerve injuries of the upper limb

With the advancements in materials science, it is anticipated that more artificial materials will be utilized to aid in the direct repair of nerve transections. Currently, the nerve conduits in use merely provide a pathway between nerve ends. It is hoped that future artificial materials will more effectively induce natural nerve regeneration across the transected ends. Secondly, regardless of the type of nerve transfer and repair technique employed, a considerable amount of time is required post-nerve suturing for the formation of neural pathways. We aspire to see more efficient methods for inducing nerve growth. Furthermore, current literature indicates that ultrasound and MRI are playing significant diagnostic and adjunctive therapeutic roles in the field of peripheral nerves. It is anticipated that in the future, ultrasound or MRI, or even more advanced diagnostic modalities, will be able to more accurately predict nerve recovery outcomes.

Lastly, the success of contralateral C7 nerve transfer has expanded the scope of nerve injury treatment from the peripheral nervous system to the central nervous system. With the ongoing exploration in nerve treatment, more patients are expected to benefit from these advancements.

### 4.5 Limitations

In this study, our analysis was confined to the Web of Science database, which may have led to the exclusion of relevant papers from other databases. Although the search strategy was designed to gather the most comprehensive dataset possible, it does not ensure that all retrieved articles are entirely pertinent to the research topic.

These limitations suggest that future studies should incorporate a broader range of databases and employ more precise analytical tools to enhance the accuracy and comprehensiveness of the research outcomes.

## Footnotes

^1^ https://www.webofscience.com/wos/woscc/basic-search

## References

[1] Spinner RJ Kline DG. Surgery for peripheral nerve and brachial plexus injuries or other nerve lesions. Muscle Nerve. (2000) 23:680–95. doi: 10.1002/(SICI)1097-4598(200005)23:5<680::AID-MUS4>3.0.CO;2-H 10797390

[2] Tung TH Mackinnon SE. Nerve transfers: indications, techniques, and outcomes. J Hand Surg. (2010) 35:332–41. doi: 10.1016/j.jhsa.2009.12.002, PMID: 20141906

[3] Giuffre JL Bishop AT Spinner RJ Shin AY. The best of tendon and nerve transfers in the upper extremity. Plast Reconstr Surg. (2015) 135:617e–30e. doi: 10.1097/PRS.0000000000001071, PMID: 25719726

[4] Harris W Low VW. On the importance of accurate muscular analysis in lesions of the brachial plexus; and the treatment of Erb’s palsy and infantile paralysis of the upper extremity by cross-union of the nerve roots. Br Med J. (1903) 2:1035–8.

[5] Gu YD Zhang GM Chen DS Yan JG Cheng XM Chen L. Seventh cervical nerve root transfer from the contralateral healthy side for treatment of brachial plexus root avulsion. J Hand Surg Br. (1992) 17:518–21. doi: 10.1016/s0266-7681(05)80235-9, PMID: 1479244

[6] Oberlin C Béal D Leechavengvongs S Salon A Dauge MC Sarcy JJ. Nerve transfer to biceps muscle using a part of ulnar nerve for C5-C6 avulsion of the brachial plexus: anatomical study and report of four cases. J Hand Surg. (1994) 19:232–7. doi: 10.1016/0363-5023(94)90011-6, PMID: 8201186

[7] Chuang DC Lee GW Hashem F Wei FC. Restoration of shoulder abduction by nerve transfer in avulsed brachial plexus injury: evaluation of 99 patients with various nerve transfers. Plast Reconstr Surg. (1995) 96:122–8. doi: 10.1097/00006534-199507000-00019, PMID: 7604091

[8] Leechavengvongs S Witoonchart K Uerpairojkit C Thuvasethakul P. Nerve transfer to deltoid muscle using the nerve to the long head of the triceps, part II: a report of 7 cases. J Hand Surg. (2003) 28:633–8. doi: 10.1016/s0363-5023(03)00199-0, PMID: 12877852

[9] Merrell GA Barrie KA Katz DL Wolfe SW. Results of nerve transfer techniques for restoration of shoulder and elbow function in the context of a meta-analysis of the English literature. J Hand Surg. (2001) 26:303–14. doi: 10.1053/jhsu.2001.21518, PMID: 11279578

[10] Garg R Merrell GA Hillstrom HJ Wolfe SW. Comparison of nerve transfers and nerve grafting for traumatic upper plexus palsy: a systematic review and analysis. J Bone Joint Surg Am. (2011) 93:819–29. doi: 10.2106/JBJS.I.01602, PMID: 21543672

[11] Witoonchart K Leechavengvongs S Uerpairojkit C Thuvasethakul P Wongnopsuwan V. Nerve transfer to deltoid muscle using the nerve to the long head of the triceps, part I: an anatomic feasibility study. J Hand Surg. (2003) 28:628–32. doi: 10.1016/s0363-5023(03)00200-4, PMID: 12877851

[12] Kim DH Cho YJ Tiel RL Kline DG. Outcomes of surgery in 1019 brachial plexus lesions treated at Louisiana State University health sciences center. J Neurosurg. (2003) 98:1005–16. doi: 10.3171/jns.2003.98.5.1005, PMID: 12744360

[13] Yang LJS Chang KWC Chung KC. A systematic review of nerve transfer and nerve repair for the treatment of adult upper brachial plexus injury. Neurosurgery. (2012) 71:417–29. doi: 10.1227/NEU.0b013e318257be98, PMID: 22811085

[14] Forli A Bouyer M Aribert M Curvale C Delord M Corcella D. Upper limb nerve transfers: a review. Hand Surg Rehabil. (2017) 36:151–72. doi: 10.1016/j.hansur.2016.11.007, PMID: 28521852

[15] Bertelli JA Ghizoni MF. Reconstruction of complete palsies of the adult brachial plexus by root grafting using long grafts and nerve transfers to target nerves. J Hand Surg. (2010) 35:1640–6. doi: 10.1016/j.jhsa.2010.06.019, PMID: 20843615

[16] Gu YD. Functional motor innervation of brachial plexus roots. An intraoperative electrophysiological study. J Hand Surg Br. (1997) 22:258–60. doi: 10.1016/s0266-7681(97)80076-9, PMID: 9150001

[17] Zheng MX Hua XY Feng JT Li T Lu YC Shen YD. Trial of contralateral seventh cervical nerve transfer for spastic arm paralysis. N Engl J Med. (2018) 378:22–34. doi: 10.1056/NEJMoa1615208, PMID: 29262271

[18] Elhassan B Bishop A Shin A Spinner R. Shoulder tendon transfer options for adult patients with brachial plexus injury. J Hand Surg. (2010) 35:1211–9. doi: 10.1016/j.jhsa.2010.05.001, PMID: 20610066

[19] Hartzler RU Barlow JD An KN Elhassan BT. Biomechanical effectiveness of different types of tendon transfers to the shoulder for external rotation. J Shoulder Elb Surg. (2012) 21:1370–6. doi: 10.1016/j.jse.2012.01.026, PMID: 22572399

[20] Ratner JA Peljovich A Kozin SH. Update on tendon transfers for peripheral nerve injuries. J Hand Surg. (2010) 35:1371–81. doi: 10.1016/j.jhsa.2010.05.023, PMID: 20684937

[21] Jones NF Machado GR. Tendon transfers for radial, median, and ulnar nerve injuries: current surgical techniques. Clin Plast Surg. (2011) 38:621–42. doi: 10.1016/j.cps.2011.07.002, PMID: 22032590

[22] Malessy MJA Bakker D Dekker AJ Van Duk JG Thomeer RTWM. Functional magnetic resonance imaging and control over the biceps muscle after intercostal-musculocutaneous nerve transfer. J Neurosurg. (2003) 98:261–8. doi: 10.3171/jns.2003.98.2.0261, PMID: 12593609

[23] Mackinnon SE Colbert SH. Nerve transfers in the hand and upper extremity surgery. Tech Hand Up Extrem Surg. (2008) 12:20–33. doi: 10.1097/BTH.0b013e31812714f3, PMID: 18388751

[24] Novak CB Mackinnon SE. Distal anterior interosseous nerve transfer to the deep motor branch of the ulnar nerve for reconstruction of high ulnar nerve injuries. J Reconstr Microsurg. (2002) 18:459–64. doi: 10.1055/s-2002-33326, PMID: 12177812

[25] Barbour J Yee A Kahn LC Mackinnon SE. Supercharged end-to-side anterior interosseous to ulnar motor nerve transfer for intrinsic musculature reinnervation. J Hand Surg. (2012) 37:2150–9. doi: 10.1016/j.jhsa.2012.07.022, PMID: 23021177

[26] Verdugo RJ Salinas RA Castillo JL Cea G. Surgical versus non-surgical treatment for carpal tunnel syndrome. Cochrane Database Syst Rev. (2008):CD001552. doi: 10.1002/14651858.CD001552.pub2 18843618 PMC7061249

[27] Hui ACF Wong S Leung CH Tong P Mok V Poon D. A randomized controlled trial of surgery vs steroid injection for carpal tunnel syndrome. Neurology. (2005) 64:2074–8. doi: 10.1212/01.WNL.0000169017.79374.93, PMID: 15985575

[28] Li Y Luo W Wu G Cui S Zhang Z Gu X. Open versus endoscopic carpal tunnel release: a systematic review and meta-analysis of randomized controlled trials. BMC Musculoskelet Disord. (2020) 21:272. doi: 10.1186/s12891-020-03306-1, PMID: 32340621 PMC7187537

[29] Yoshii Y Zhao C Amadio PC. Recent advances in ultrasound diagnosis of carpal tunnel syndrome. Diagnostics (Basel). (2020) 10:596. doi: 10.3390/diagnostics10080596, PMID: 32824261 PMC7460039

[30] Ng AWH Griffith JF Tong CSL Law EKC Tse WL Wong CWY. MRI criteria for diagnosis and predicting severity of carpal tunnel syndrome. Skeletal Radiol. (2020) 49:397–405. doi: 10.1007/s00256-019-03291-0, PMID: 31396669

[31] Padua L Cuccagna C Giovannini S Coraci D Pelosi L Loreti C. Carpal tunnel syndrome: updated evidence and new questions. Lancet Neurol. (2023) 22:255–67. doi: 10.1016/S1474-4422(22)00432-X, PMID: 36525982

[32] Rydberg M Perez R Merlo J Dahlin LB. Carpal tunnel syndrome and trigger finger may be an early symptom of Preclinic type 2 diabetes. Plast Reconstr Surg Glob Open. (2024) 12:e5907. doi: 10.1097/GOX.0000000000005907, PMID: 38881965 PMC11177834

[33] Mackinnon SE Novak CB Myckatyn TM Tung TH. Results of reinnervation of the biceps and brachialis muscles with a double fascicular transfer for elbow flexion. J Hand Surg Am. (2005) 30:978-985. doi: 10.1016/j.jhsa.2005.05.014 16182054

